# A Comparative Study of Generic Visual Components of Two-Dimensional Versus Three-Dimensional Laparoscopic Images

**DOI:** 10.1007/s00268-017-4220-3

**Published:** 2017-09-21

**Authors:** Michael El Boghdady, Gobinath Ramakrishnan, Benjie Tang, Afshin Alijani

**Affiliations:** Cuschieri Skills Centre, Ninewells Hospital and Medical School, University of Dundee, Dundee, DD19SY UK

## Abstract

**Aims:**

There is a strong evidence to suggest that 3D imaging improves the laparoscopic task performance when compared against 2D. However, to date, no study has explained why that might be. We identified six generic visual components during laparoscopic imaging and aimed to study each component in both 2D and 3D environments for comparison.

**Methods:**

Twenty-four consented laparoscopic novices performed specific isolated tasks in a laparoscopic Endo Trainer in 2D and 3D separately. The six endpoints were the accuracy in detecting changes in the laparoscopic images in the following components: distance, area, angle, curvature, volume and spatial coordinates. All the components except the spatial coordinates were assessed by creation, measurement and comparison. Each component was analysed between 2D and 3D groups and within each group at different values. Tests of spatial coordinates were video-recorded and analysed for error number and error types by human reliability analysis technique. Errors types included past-pointing, not reaching the object and touching the wrong object. The results were statistically analysed with independent *T* test.

**Results:**

There was no statistically significant difference between 2D and 3D accuracy in the angle, area, distance and curvature. 3D performed more accurately in comparing volumes (*p* = 0.05). In spatial coordinates, there were a statistically significant higher number of errors in 2D as compared to 3D (*p* < 0.001). Past-pointing and touching the wrong objects were significantly higher in 2D (*p* < 0.05).

**Conclusion:**

Between all the visual components, detecting change in volume and the spatial coordinates showed significant improvement in 3D environment when compared to 2D.

## Introduction

There is a strong evidence to suggest that three-dimensional (3D) imaging improves the surgical task performance during laparoscopic surgery [1, 2]. However, to date, no study has explained the reasons behind this apparent improvement in the 3D environment. To understand what components of the 3D image affect the task performance, we identified six generic visual components of any laparoscopic image and aimed to study each component in both 2D and 3D environments for comparison.

## Methods

Consented laparoscopic novices from medical students were included in this study. Each participant was randomly crossed over between 2D and 3D imaging. The participants took part in a battery of tests (Table 1), conducted in a laparoscopic Endo Trainer (Body Torso Simulator box, Pharmabotics Ltd, Hampshire) and using a laparoscope (26003BA, Hopkins^®^, 30°, 10 mm diameter, 31 cm length, Karl Storz) with HD 2D and HD 3D systems (19 inch, resolution 1920 × 1080 pixels, Karl Storz GmbH & Co, Tuttlingen, Germany). The optimal distance between the end of the endoscope and the target was standardized at 10 cm, and the distance between each participant and the screen was set at 1 m [3]. The port was inserted into the Endo Trainer to create a 90° angle between the image axis and the target.

**Table 1:** Component tests

Component	Creation	Comparison	Measurement
Distance Ref—1.5 cm	To create a distance of 2/3.5/4.5/6 cm	To compare distance of 4.0/4.15/4.30/4.45 cm	To measure a given distance 4/6/7/9 cm
Area Ref—1.5 cm	Omitted due to task complexity	To compare areas of different circle—within 0.15 cm/0.2 cm increments Circle—4/4.2/4.4/4.6 cm (diameter)	To measure area of given circle Circle—5/6/7/9 cm (diameter)
Angle Ref—15°	To create following random angle 5°/30°/50°	To compare different angle 30°/32°/34°/36°/38° (the sides of each angle will be 4 cm in length, 3 mm width)	To measure the following drawn angles one at a time 25/35/45/65
Curvature	Omitted due to task complexity	To compare a curvature The curvature is created with changing the radius from 3/4/5/6 cm	Omitted due to task complexity
Volume Ref—2 ml	To create a volume by injecting Foley’s balloon catheter Volume—3/5/8 ml	To compare volumes of different balloon 3/4/5/8 ml	To measure the given volume 3/5/7 ml

Five generic components of the laparoscopic image of an object were identified. These included distance, area, angle, curvature and volume. Each component was isolated and studied independently in both 2D and 3D laparoscopic environments for comparison. The study of the spatial coordinates of objects in the laparoscopic environment was also included as a global test comparing the task performance in 2D versus 3D environments. Each of the five generic components except spatial coordinate test was assessed by the method of measuring, comparing and creating. The measurement task tested the ability of the participant to estimate a given measurement in any of the components. The comparison task assessed how the participants could compare the given components of varying measurements. The creation task involved the ability of the participant to create a given measurement in selected components (distance, angle and volume). Each component test took approximately 15 s to complete.

For distance, participants were asked to create a predefined length by using a laparoscopic grasper to move a referenced peg along a string. The length created was then measured from the end of the string to the placed peg. Subjects were asked to compare and measure standardized distances separately (Table 1).

**Fig. 1:**
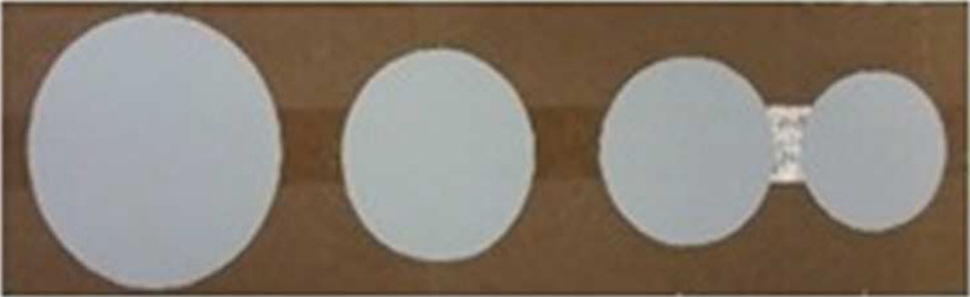
Circle area comparison

Circles with different diameters were placed alongside each other, and the participants were asked to place them laparoscopically in the order of increasing area (Fig. 1). For area measurement, subjects were asked to estimate the area of given circles (Table 1). Area creation was excluded due to the complexity of the test.

**Fig. 2:**
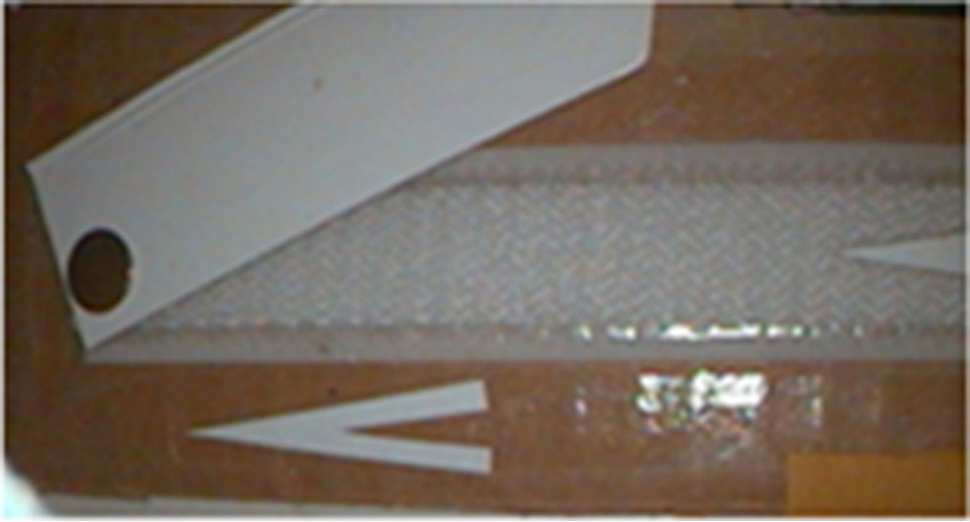
Angle creation

**Fig. 3:**
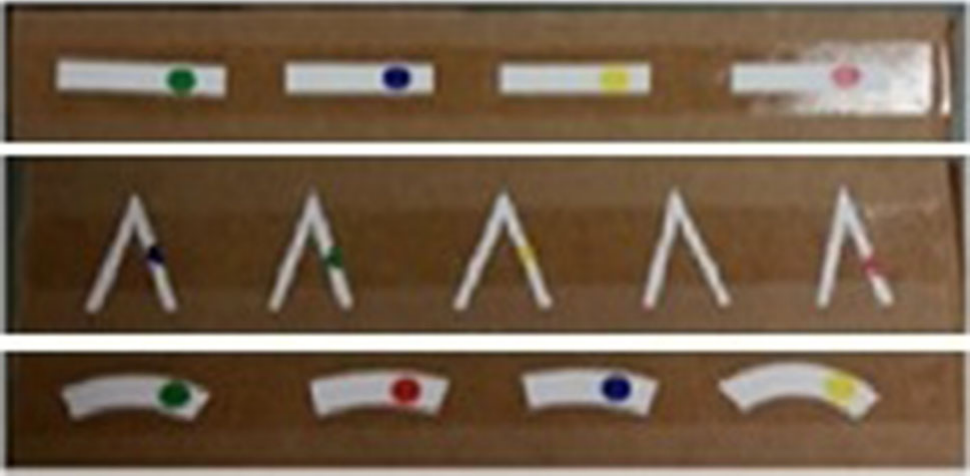
Distance, angle and curvature comparison

For angle, subjects created different predefined angles using a laparoscopic grasper by moving an adjustable arm attached to a fixed horizontal arm with a hinged vertex (Fig. 2). Each angle created was measured separately. The comparison test involved pieces of paper which had been cut according to the different angles and were labelled with various colours for the identification (Fig. 3). Subjects were asked to estimate four different standardized angles separately (Table 1).

Curvature comparison was performed by using pieces of paper which had been cut according to different curvatures from the circumference of different sized circles and were labelled with various colours for the identification (Table 1; Fig. 3). Curvature creation and measurement were excluded due to the complexity of tests.

For volume, participants were asked to create different predefined volumes in the shape of a sphere by instructing another person to inject air into the balloon of a Foley’s catheter viewed laparoscopically. Subjects were given an appropriate reference scale to help them with the creation task. For volume measurement, participants used a syringe with predefined volumes of air and participants were asked to put the shapes of different volumes in order of size. Participants were presented with different predefined volumes of inflated Foley’s catheter balloon and were asked to estimate each volume (Table 1). In the comparison tests, the number of sequence which was guessed correctly was calculated.

**Fig. 4:**
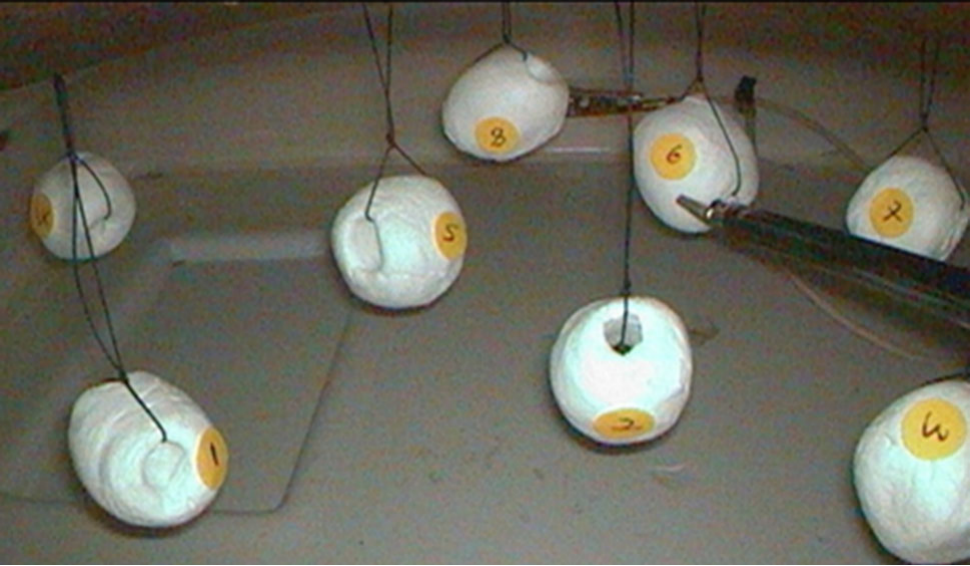
Spatial coordinates test

In the spatial coordinates, eight numbered small clay balls were suspended from the top of a pelvis of a laparoscopic Endo Trainer, using strings at different spatial coordinates (Fig. 4). Each participant was required to touch the objects laparoscopically using a grasper in 2D and 3D imaging following a set of predefined rules. The rules were as follows: using dominant hand, touching fixed random sequence objects alternately (objects 1, 3, 5, 7 and objects 2, 4, 6, 8), avoid touching other objects or strings and completing the task within 1 min. The endpoints for the spatial coordinates test were the errors committed (type and number of error), number of movements and the number of objects that the participant could touch correctly within the 1 min given. Errors in spatial coordinates test were identified as: past-pointing, not reaching the object and touching the wrong object. The endpoints of spatial coordinate test were type and total number of errors, the number of instrument movements and number of objects that were correctly touched.

The results were analysed with IBM SPSS version 22. The recorded laparoscopic videos of spatial coordinate test were analysed with human reliability analysis [4]. Paired *t* test was used to detect any significant difference (assumed normal distribution). A *p* value less than 0.05 was accepted as statistically significant.

## Results

**Fig. 5:**
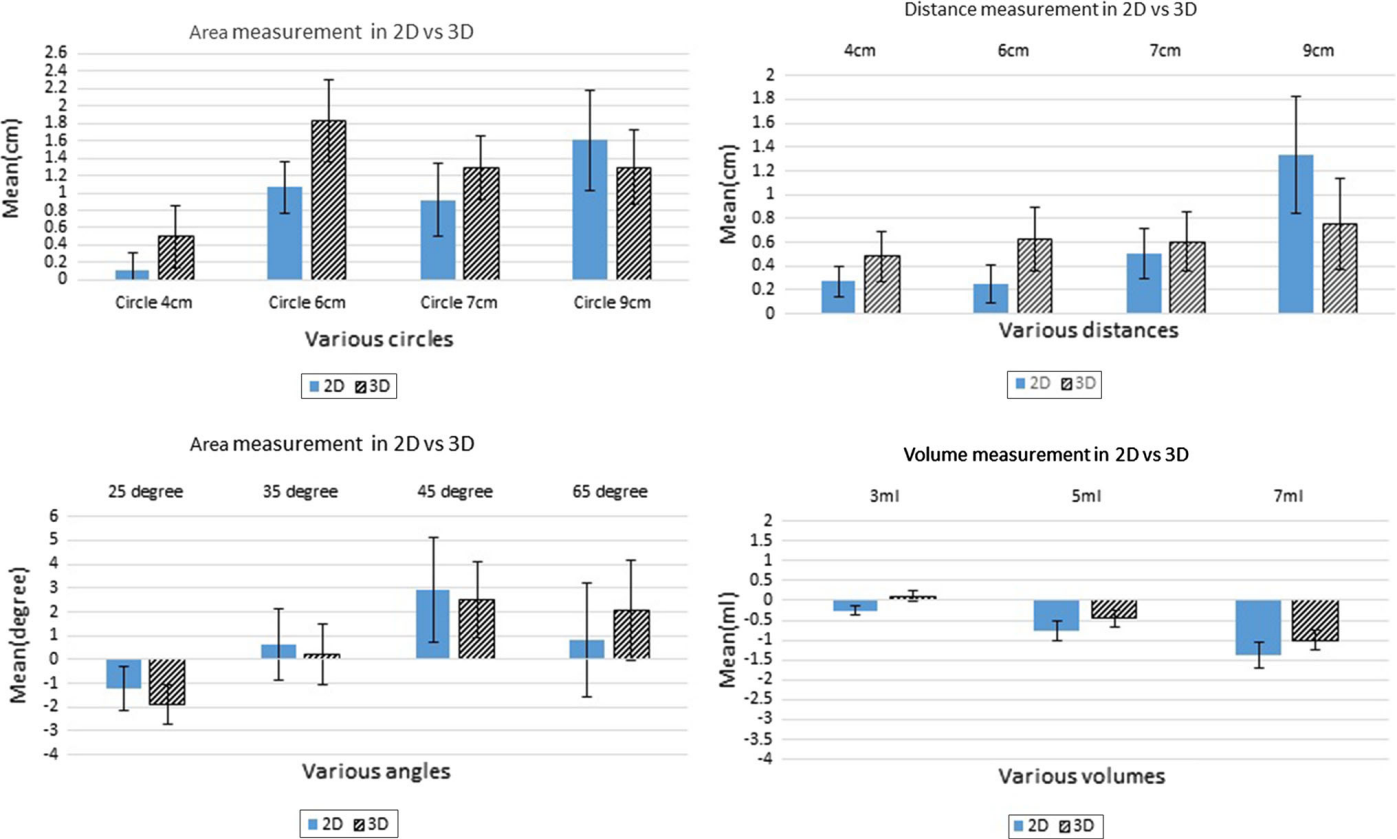
Results of area, distance, angle and volume measurement

**Fig. 6:**
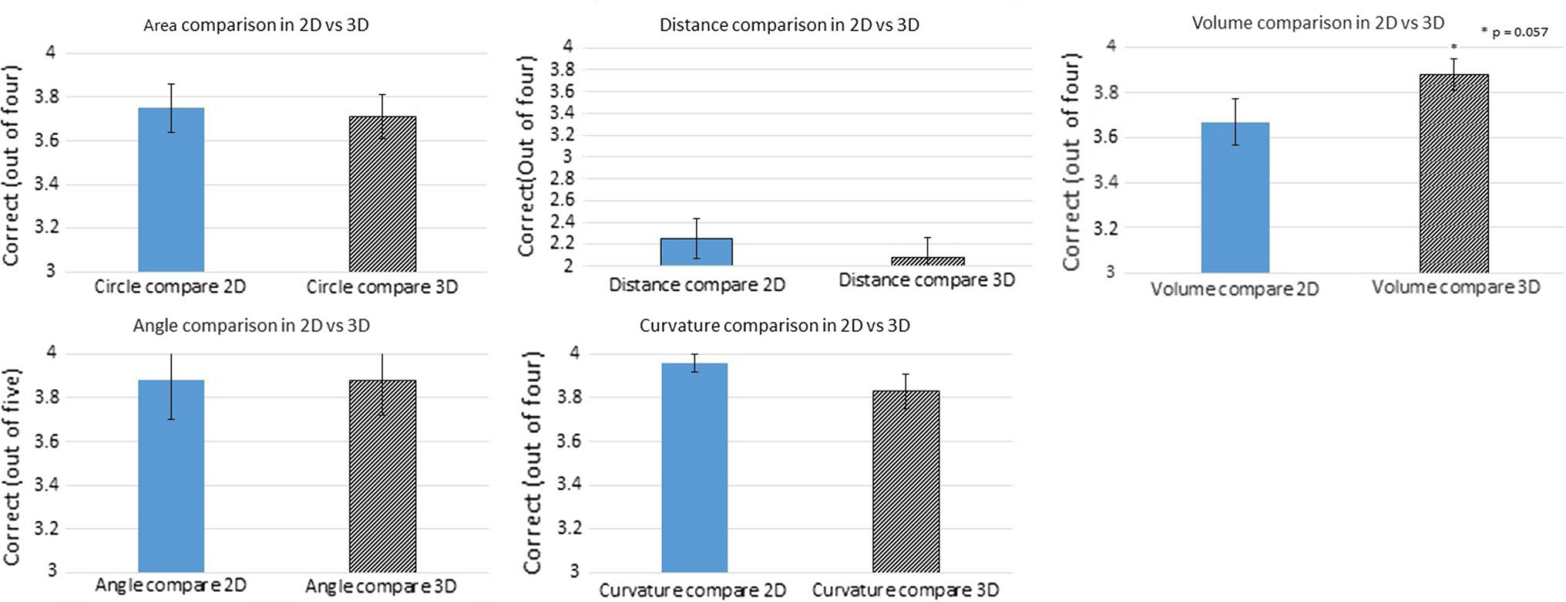
Results of area, distance, angle, curvature and volume comparison

**Fig. 7:**
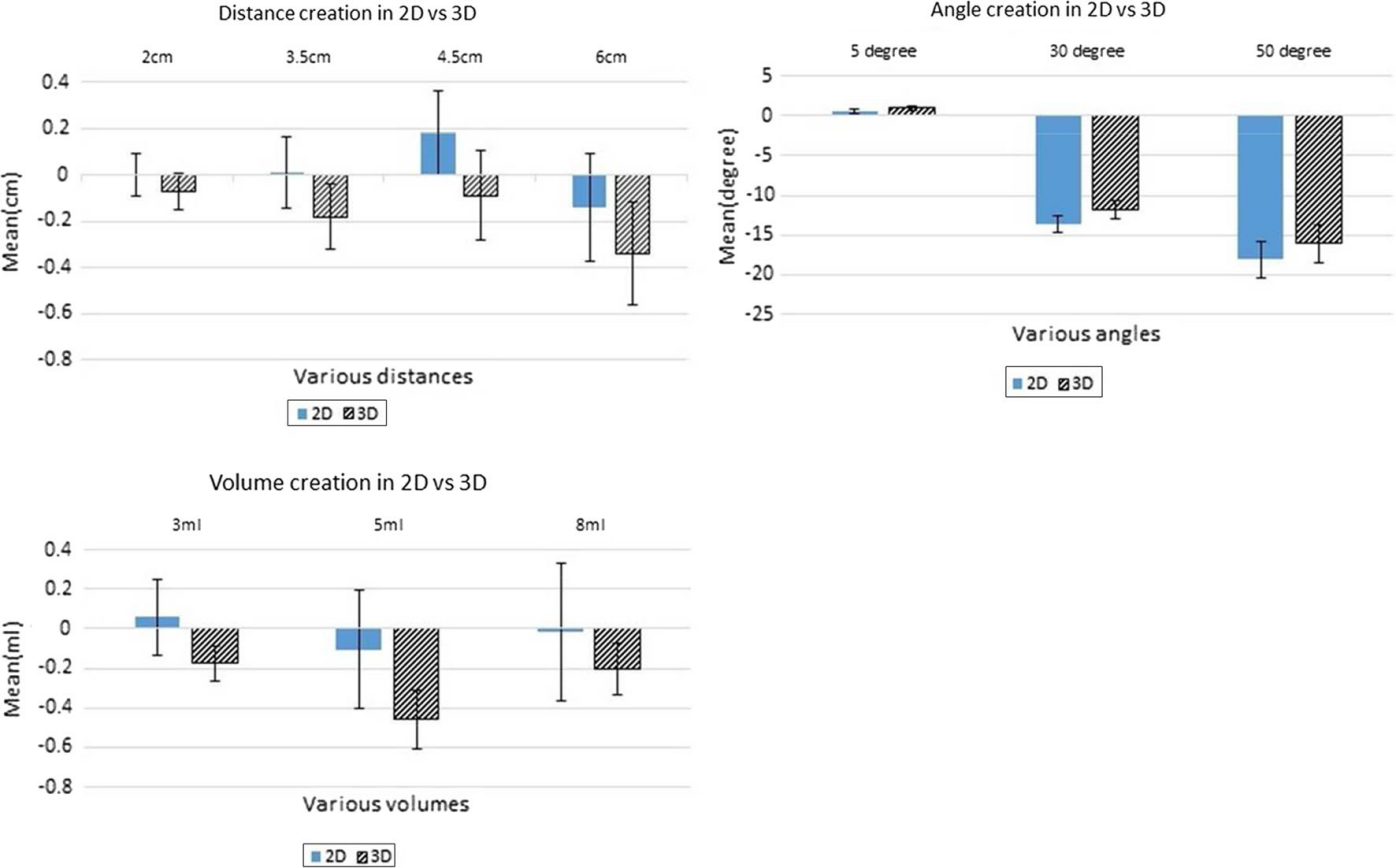
Results of distance, angle and volume creation

Twenty-four medical students participated in this study. In the measurement of volumes, 3D did better than 2D at 3 ml. However, there was no difference at 5 and 7 ml between the two groups (Fig. 5). There was a statistical difference in volume comparison, with 3D showing superiority compared to 2D (*p* = 0.057) (Fig. 6). For volume creation, 2D imaging showed more uncertainties with wider confidence interval compared to 3D. However, the difference between the 2D and 3D was not significant (Fig. 7). There was a trend of underestimation of volume measurement with 3D showing more accuracy.

**Fig. 8:**
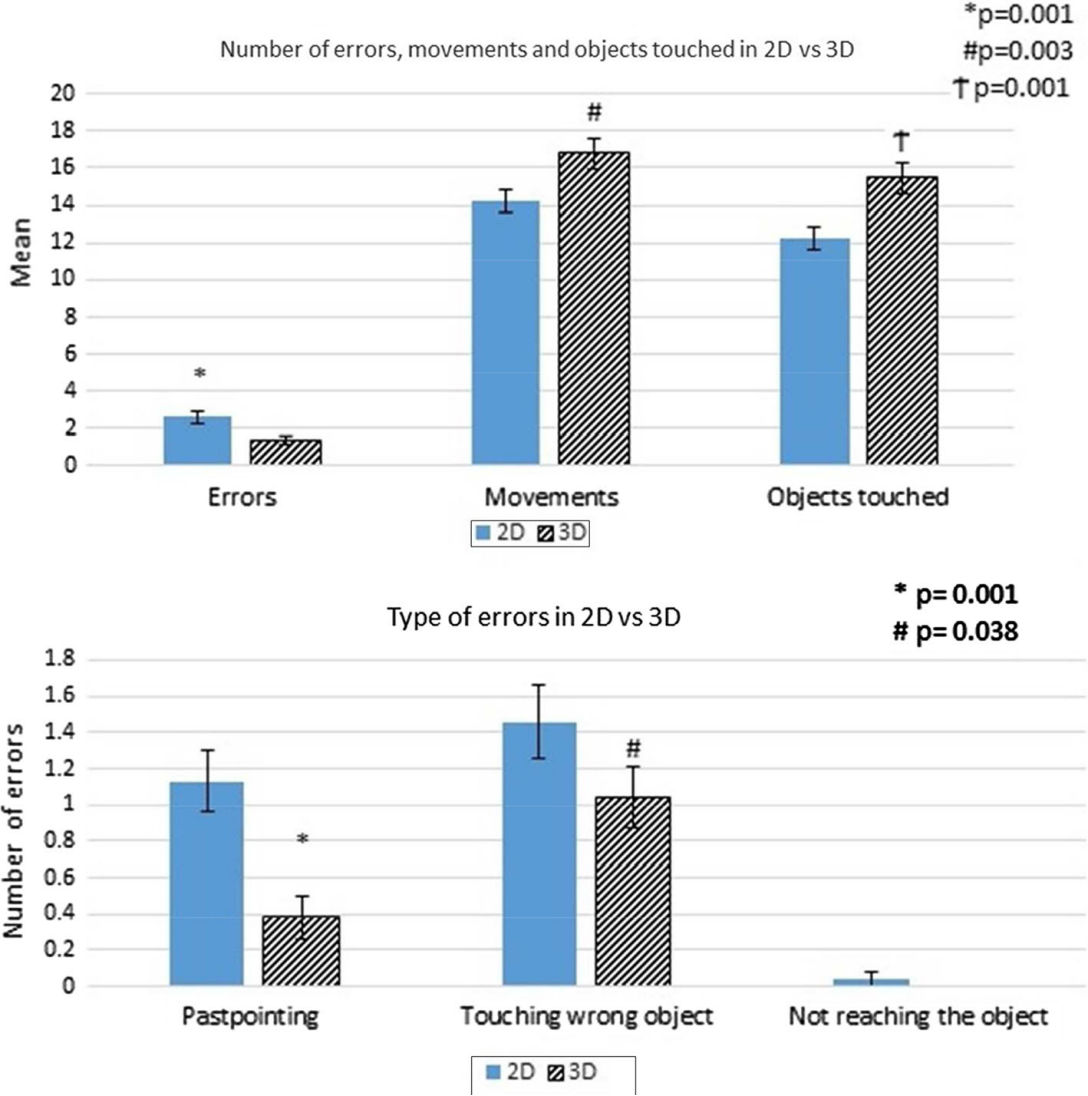
Results of the spatial coordinates tests

For the spatial coordinates test, there were a statistically significant higher number of errors in 2D imaging (*p* = 0.001). For the type of errors, the past-pointing (*p* = 0.001) and touching wrong objects (*p* = 0.038) were statistically significant and higher in 2D (Fig. 8). For the number of objects that could be touched within a minute, the 3D imaging performed better with a statistically significant value (*p* = 0.001) (Fig. 8).

There was no statistically significant difference between 2D and 3D accuracy in the angle, area, distance and curvature.

## Discussion

We have shown that 3D imaging improves the task performance in detecting change in volume and in spatial coordinates when compared to 2D. There was no statistical difference in detecting changes in the area, angle, distance and curvature between 2D and 3D surgical imaging.

This is the first attempt studying the individual visual components of a laparoscopic image in 2D and 3D laparoscopy. The task performance and surgical errors have previously been assessed by using composite tests called the fundamentals of laparoscopic modules (peg transfer, precision cutting, ligating loop, extra-corporeal knotting and intra-corporeal knotting) [5]. Unlike the methods used in our study, these tests consist of interplay of various dimensions and are not testing any aspect in isolation.

Many studies have shown that 3D laparoscopy improves the task performance when compared to 2D [1, 2]. Learning of laparoscopic skills involves hand–eye coordination, manual dexterity and visual spatial coordination. Unlike open surgery, in the conventional 2D laparoscopic image, the surgeon requires to interpret the image into a 3D imagery [6]. This is made more difficult by a narrow working space, magnification and pressure of acquiring new skills. In 3D laparoscopy, the surgeon adjusts artificial 3D imagery to self-constructed 3D view. The 3D laparoscopic image requires less mental processing than a 2D for constructing a realistic image in a surgeon’s mind. This could explain partly why 3D imaging improves surgical task performance.

There are a number of basic physical characteristic of any shape of image, which consists of distance, area, angle, curvature and volume. While the distance and curvature are one-dimensional, the area and angle are two-dimensional, and volume is in form of three-dimensional in character. A further factor is the position of the shape or object in space or in relation to the surrounding structures, we called this spatial coordinates.

Depth perception is the visual ability to perceive the distance of an object to a reference point. Depth perception, size and distance are ascertained through both monocular and binocular cues. Monocular vision is known to be poor at determining depth. The tests for depth perception in our study were past-pointing and the number of movements. All participants had no ocular conditions that may reduce the perception of depth such as amblyopia and strabismus. Detecting the ability of touching objects was tested by detecting errors in touching the wrong objects. The results showed that 3D images detect depth perception better when compared to 2D.

The ability to measure distance is essential as the surgeon has to estimate the distance of the crucial structures in the working area, for example, the positioning of the tip of the needle into the tissue during continuous running suture to create equal distance sutures. A surgeon should be competent in measuring and estimating distance. This quality is compromised in conventional 2D laparoscopic system due to the image magnification. We have shown that 3D imaging does not give any advantage in detecting change in distance when compared to 2D. This is an important factor to bear in mind when using a 3D laparoscopy that the task components of the laparoscopic image are not improved using 3D.

An example of estimating the area during surgery is the laparoscopic mesh repair of the groin, incisional and ventral hernias. In general, estimating the diameter of a circle is the visual cue for appreciating its area. We have shown that 3D imaging does not give any advantage in appreciating the area when compared to 2D laparoscopy.

We also have shown that 3D laparoscopy does not improve the appreciation of changes in the angle component of the image. A practical example of angle and its appreciation in surgery can be seen in the ability of the surgeon to place and adjust a suture needle at the desired angle to the needle holder. Another example will be the adjustment of the angle of the roticulating laparoscopic stapler.

The curvature of a circle is the inverse of its radius. Small radius creates sharp curve, and large radius will create a smoother curve. Most anatomical structures have a curvature. Appreciating the curvature of the structures is important in laparoscopic surgery, for grasping the fundus of the gall bladder at the appropriate place for retraction during the dissection of Calot’s triangle in laparoscopic cholecystectomy is a good example in appreciating the importance of curvature. In our study, 3D imaging does not provide any advantage over 2D in curvature.

3D laparoscopy improved participants’ detection of change in volume in this study. The ability to estimate volume accurately has many uses in laparoscopic surgery. An example of this is when the attending surgeon needs to create a gastric pouch during bariatric surgery.

A further experiment was conducted to compare 2D to 3D in locating the position of objects in space. The previous experiments (distance, curvature, angle and volume) studied the characters of objects itself; the spatial coordinates experiment tested the ability to judge the location of the object in relation to the surrounding environment. This showed that 3D imaging provided a clear advantage over 2D with regards to the spatial coordinates of the object.

The findings of this study might be relevant when it comes to future design of software programming and algorithms, putting in mind that 3D imaging shows difference in volume and spatial coordinates, and not in the distance, area, angle and curvature.

## Conclusion

Between all the visual components, detecting change in volume and the spatial coordinates showed significant improvement in 3D environment when compared to 2D.
